# Crystal structure of 3-{(*E*)-[(3,4-di­chloro­phen­yl)imino]­meth­yl}benzene-1,2-diol

**DOI:** 10.1107/S2056989015001401

**Published:** 2015-01-28

**Authors:** Muhammad Nawaz Tahir, Hazoor Ahmad Shad, Abdul Rauf, Abdul Haleem Khan

**Affiliations:** a Department of Physics, University of Sargodha, Sargodha, Punjab, Pakistan; b Department of Chemistry, University of Sargodha, Sargodha, Pakistan; c Department of Chemistry, Chenab College, Jhang, Punjab, Pakistan; d Drug Controller, Sir Ganga Ram Hospital, Lahore, Pakistan

**Keywords:** crystal structure, benzene-1,2-diol, Schiff base, hydrogen bonding

## Abstract

In the title Schiff base, C_13_H_9_Cl_2_NO_2_, which arose from the condensation of 3,4-di­chloro­aniline with 2,3-di­hydroxy­benzaldehyde, the dihedral angle between the aromatic rings is 44.74 (13)°. Intra­molecular O—H⋯O and O—H⋯N hydrogen bonds close *S*(5) and *S*(6) rings, respectively. In the crystal, inversion dimers linked by pairs of O—H⋯O hydrogen bonds generate *R*_2_^2^(10) loops. A weak C—H⋯π inter­action is also observed.

## Related literature   

For related structures, see: Fun *et al.* (2011 ▸); Keleşoğlu *et al.* (2009 ▸); Shuja *et al.* (2006 ▸); Tahir *et al.* (2012 ▸); Temel *et al.* (2007 ▸).
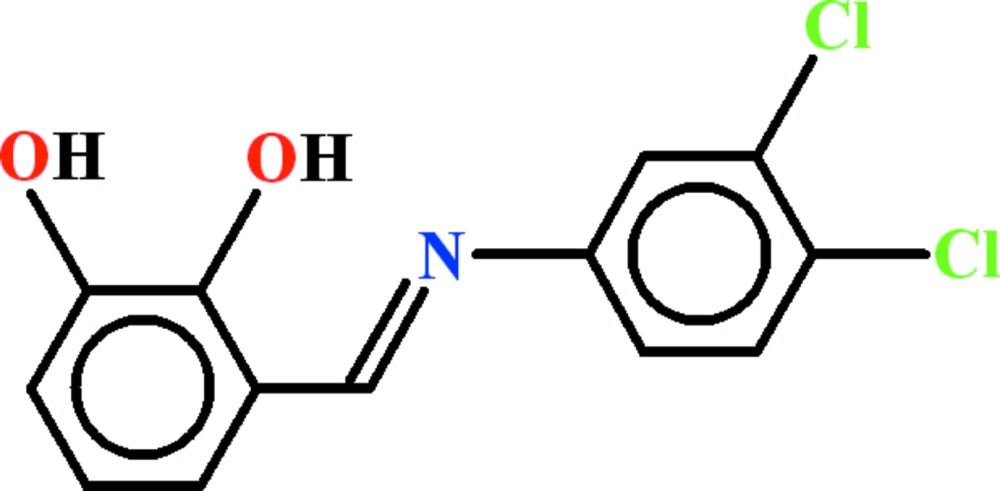


## Experimental   

### Crystal data  

C_13_H_9_Cl_2_NO_2_
*M* *_r_* = 282.11
Triclinic, 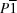

*a* = 6.4237 (8) Å
*b* = 8.8412 (11) Å
*c* = 11.7799 (15) Å
α = 88.606 (6)°
β = 76.588 (6)°
γ = 70.193 (5)°
*V* = 611.20 (13) Å^3^
*Z* = 2
Mo *K*α radiation
μ = 0.52 mm^−1^
*T* = 296 K
0.34 × 0.26 × 0.20 mm

### Data collection  

Bruker Kappa APEXII CCD diffractometer
Absorption correction: multi-scan (*SADABS*; Bruker, 2005 ▸) *T*_min_ = 0.844, *T*_max_ = 0.902
8896 measured reflections
2671 independent reflections
1866 reflections with *I* > 2σ(*I*)
*R*_int_ = 0.042

### Refinement  

*R*[*F* ^2^ > 2σ(*F* ^2^)] = 0.052
*wR*(*F* ^2^) = 0.164
*S* = 1.04
2671 reflections
165 parameters
H-atom parameters constrained
Δρ_max_ = 0.33 e Å^−3^
Δρ_min_ = −0.33 e Å^−3^

### 

Data collection: *APEX2* (Bruker, 2007 ▸); cell refinement: *SAINT* (Bruker, 2007 ▸); data reduction: *SAINT*; program(s) used to solve structure: *SHELXS97* (Sheldrick, 2008 ▸); program(s) used to refine structure: *SHELXL97* (Sheldrick, 2015 ▸); molecular graphics: *ORTEP-3 for Windows* (Farrugia, 2012 ▸) and *PLATON* (Spek, 2009 ▸); software used to prepare material for publication: *WinGX* (Farrugia, 2012 ▸) and *PLATON*.

## Supplementary Material

Crystal structure: contains datablock(s) global, I. DOI: 10.1107/S2056989015001401/hb7353sup1.cif

Structure factors: contains datablock(s) I. DOI: 10.1107/S2056989015001401/hb7353Isup2.hkl

Supporting information file. DOI: 10.1107/S2056989015001401/hb7353Isup3.cml

**Fig. 1.**
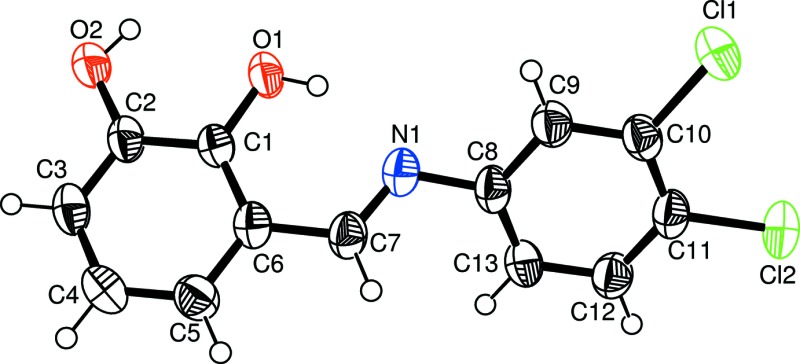
View of the title compound with displacement ellipsoids drawn at the 50% probability level.

. DOI: 10.1107/S2056989015001401/hb7353fig1.tif
View of the title compound with displacement ellipsoids drawn at the 50% probability level.

**Fig. 2.**
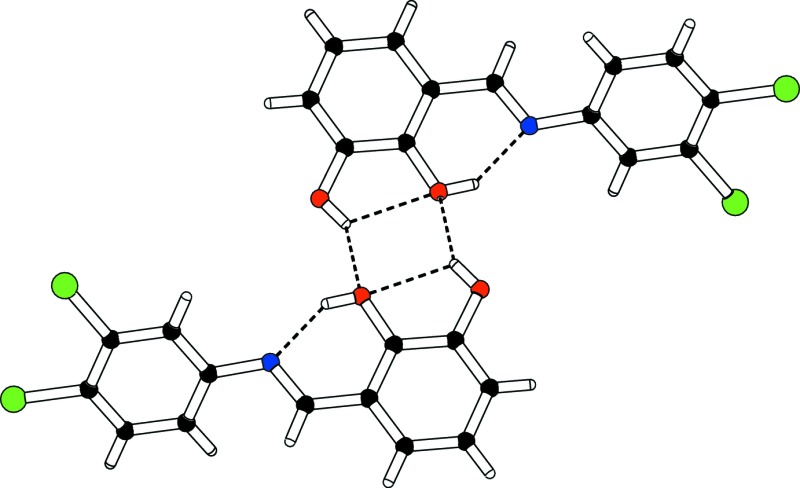
The partial packing (PLATON; Spek, 2009), which shows that molecules form dimers due to O—H···O interactions.

PLATON . DOI: 10.1107/S2056989015001401/hb7353fig2.tif
The partial packing (*PLATON*; Spek, 2009), which shows that mol­ecules form dimers due to O—H⋯O inter­actions.

CCDC reference: 1044861

Additional supporting information:  crystallographic information; 3D view; checkCIF report

## Figures and Tables

**Table 1:** Hydrogen-bond geometry (, ) *Cg*1 is the centroid of the benzene ring (C1C6).

*D*H*A*	*D*H	H*A*	*D* *A*	*D*H*A*
O1H1N1	0.82	1.89	2.608(3)	146
O2H2O1	0.82	2.28	2.729(3)	115
O2H2O1^i^	0.82	2.20	2.846(3)	136
C12H12*Cg*1^ii^	0.93	2.83	3.538(4)	134

Symmetry codes: (i) 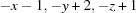; (ii) 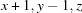.

## supplementary crystallographic information

### S1. Comment

The title compound (I), (Fig. 1) has been synthesized for forming different metal complexes.

The crystal structures of 2-[(*E*)-(2,4,6-trichlorophenyl)iminomethyl] phenol (Fun *et al.*, 2011), (*E*)-3-((2-fluorophenylimino)methyl) benzene-1,2-diol (Temel *et al.*, 2007), (*E*)-3-[(3-bromophenyl) iminomethyl]benzene-1,2-diol (Kelesoglu *et al.*, 2009), 4-([(*E*)-2, 3-dihydroxybenzylidene]amino)-*N*-(5-methyl-1,2-oxazol-3-yl)benzenesulfonamide (Tahir *et al.*, 2012) and 3-(4-bromophenyliminomethyl)benzene-1,2-diol (Shuja *et al.*, 2006) have been published which are related to the title compound due to two moieties of the Schiff base.

In (I) the moieties of 2,3-dihydroxybenzaldehyde A (C1–C7/O1/O2) and 3,4-dichloroanilline B (C8—13/N1/CL1/CL2) are almost planar with r.m.s. deviation of 0.0225 and 0.0172 Å, respectively. The dihedral angle between A/B is 44.219 (50)°. In (I), *S*(5) and *S*(6) ring motifs are present due to H-bondings of O—H···O and O—H···N types (Table 1, Fig. 2). The molecules are dimerized due to bifercated H-bonding of O—H···O type (Table 1, Fig. 2). There exist C—H···π (Table 1) and π···π interactions to stablize the dimmers. A π···π interactions between *Cg*1···*Cg*1^i^ [i = -*x*, 2 - *y*, 1 - *z*] at a distance of 3.9101 (15) Å, where *Cg*1 is centroid of benzene ring (C1—C6) exists. Similarly, there is π···π interactions between *Cg*2···*Cg*2^ii^ [il = 1 - *x*, 1 - *y*, - *z*] at a distance of 4.1194 (17) Å, where *Cg*2 is centroid of benzene ring (C8—C13).

### S2. Experimental

Equimolar quantities of 3,4-dichloroanilline and 2,3-dihydroxybenzaldehyde were refluxed in methanol for 2 h. The resulting mixture was evaporated to grow crystals. Red prisms were obtained after 48 h.

### S3. Refinement

The H-atoms were positioned geometrically (C–H = 0.93 Å, O—H = 0.82 Å) and refined as riding with *U*_iso_(H) = *xU*_eq_(C, O), where *x* = 1.5 for hydroxy and *x* = 1.2 for other H-atoms.

### Crystal data

**Table:**

C_13_H_9_Cl_2_NO_2_	*Z* = 2
*M**_r_* = 282.11	*F*(000) = 284
Triclinic, *P*1	*D*_x_ = 1.533 Mg m^−^^3^
*a* = 6.4237 (8) Å	Mo *K*α radiation, λ = 0.71073 Å
*b* = 8.8412 (11) Å	Cell parameters from 1866 reflections
*c* = 11.7799 (15) Å	θ = 1.8–27.0°
α = 88.606 (6)°	µ = 0.52 mm^−^^1^
β = 76.588 (6)°	*T* = 296 K
γ = 70.193 (5)°	Prism, red
*V* = 611.20 (13) Å^3^	0.34 × 0.26 × 0.20 mm

### Data collection

**Table:**

Bruker Kappa APEXII CCD diffractometer	2671 independent reflections
Radiation source: fine-focus sealed tube	1866 reflections with *I* > 2σ(*I*)
Graphite monochromator	*R*_int_ = 0.042
Detector resolution: 7.80 pixels mm^-1^	θ_max_ = 27.0°, θ_min_ = 1.8°
ω scans	*h* = −8→7
Absorption correction: multi-scan (*SADABS*; Bruker, 2005)	*k* = −11→9
*T*_min_ = 0.844, *T*_max_ = 0.902	*l* = −14→15
8896 measured reflections	

### Refinement

**Table:**

Refinement on *F*^2^	Primary atom site location: structure-invariant direct methods
Least-squares matrix: full	Secondary atom site location: difference Fourier map
*R*[*F*^2^ > 2σ(*F*^2^)] = 0.052	Hydrogen site location: inferred from neighbouring sites
*wR*(*F*^2^) = 0.164	H-atom parameters constrained
*S* = 1.04	*w* = 1/[σ^2^(*F*_o_^2^) + (0.0781*P*)^2^ + 0.3755*P*] where *P* = (*F*_o_^2^ + 2*F*_c_^2^)/3
2671 reflections	(Δ/σ)_max_ < 0.001
165 parameters	Δρ_max_ = 0.33 e Å^−^^3^
0 restraints	Δρ_min_ = −0.32 e Å^−^^3^

### Special details

**Table:**

Geometry. All e.s.d.'s (except the e.s.d. in the dihedral angle between two l.s. planes) are estimated using the full covariance matrix. The cell e.s.d.'s are taken into account individually in the estimation of e.s.d.'s in distances, angles and torsion angles; correlations between e.s.d.'s in cell parameters are only used when they are defined by crystal symmetry. An approximate (isotropic) treatment of cell e.s.d.'s is used for estimating e.s.d.'s involving l.s. planes.
Refinement. Refinement of *F*^2^ against ALL reflections. The weighted *R*-factor *wR* and goodness of fit *S* are based on *F*^2^, conventional *R*-factors *R* are based on *F*, with *F* set to zero for negative *F*^2^. The threshold expression of *F*^2^ > σ(*F*^2^) is used only for calculating *R*-factors(gt) *etc*. and is not relevant to the choice of reflections for refinement. *R*-factors based on *F*^2^ are statistically about twice as large as those based on *F*, and *R*-factors based on ALL data will be even larger.

### Fractional atomic coordinates and isotropic or equivalent isotropic displacement parameters (Å^2^)

**Table:**

	*x*	*y*	*z*	*U*_iso_*/*U*_eq_	
Cl1	0.24642 (15)	0.26936 (10)	0.01956 (8)	0.0657 (3)	
Cl2	0.73742 (15)	0.11968 (10)	0.06575 (9)	0.0707 (3)	
O1	−0.2559 (3)	0.9592 (2)	0.38833 (19)	0.0508 (5)	
H1	−0.1671	0.8794	0.3473	0.076*	
O2	−0.4973 (3)	1.2453 (3)	0.5138 (2)	0.0594 (6)	
H2	−0.5353	1.1679	0.5037	0.089*	
N1	0.1345 (4)	0.7735 (3)	0.2617 (2)	0.0452 (6)	
C1	−0.1526 (5)	1.0707 (3)	0.3887 (2)	0.0388 (6)	
C2	−0.2793 (5)	1.2167 (3)	0.4525 (2)	0.0440 (6)	
C3	−0.1834 (5)	1.3338 (3)	0.4543 (2)	0.0478 (7)	
H3	−0.2694	1.4313	0.4964	0.057*	
C4	0.0401 (5)	1.3083 (4)	0.3943 (3)	0.0516 (7)	
H4	0.1030	1.3886	0.3954	0.062*	
C5	0.1694 (5)	1.1613 (4)	0.3322 (3)	0.0494 (7)	
H5	0.3201	1.1427	0.2932	0.059*	
C6	0.0738 (5)	1.0419 (3)	0.3285 (2)	0.0409 (6)	
C7	0.2124 (5)	0.8875 (3)	0.2674 (2)	0.0440 (6)	
H7	0.3638	0.8708	0.2309	0.053*	
C8	0.2859 (5)	0.6190 (3)	0.2123 (2)	0.0433 (6)	
C9	0.2070 (5)	0.5315 (3)	0.1462 (2)	0.0464 (7)	
H9	0.0614	0.5751	0.1334	0.056*	
C10	0.3479 (5)	0.3784 (3)	0.0998 (2)	0.0447 (6)	
C11	0.5634 (5)	0.3123 (3)	0.1205 (2)	0.0471 (7)	
C12	0.6396 (5)	0.3969 (4)	0.1879 (3)	0.0490 (7)	
H12	0.7834	0.3514	0.2027	0.059*	
C13	0.5000 (5)	0.5513 (3)	0.2339 (2)	0.0480 (7)	
H13	0.5511	0.6091	0.2793	0.058*	

### Atomic displacement parameters (Å^2^)

**Table:**

	*U*^11^	*U*^22^	*U*^33^	*U*^12^	*U*^13^	*U*^23^
Cl1	0.0749 (6)	0.0607 (5)	0.0663 (5)	−0.0278 (4)	−0.0167 (4)	−0.0188 (4)
Cl2	0.0675 (6)	0.0459 (5)	0.0815 (6)	−0.0049 (4)	−0.0040 (4)	−0.0243 (4)
O1	0.0505 (11)	0.0377 (10)	0.0591 (13)	−0.0162 (9)	0.0005 (9)	−0.0157 (9)
O2	0.0502 (12)	0.0463 (12)	0.0765 (15)	−0.0184 (10)	0.0001 (11)	−0.0216 (11)
N1	0.0488 (13)	0.0387 (12)	0.0405 (12)	−0.0089 (10)	−0.0041 (10)	−0.0057 (10)
C1	0.0451 (14)	0.0348 (13)	0.0361 (13)	−0.0133 (11)	−0.0086 (11)	−0.0023 (10)
C2	0.0503 (16)	0.0365 (14)	0.0419 (14)	−0.0111 (12)	−0.0094 (12)	−0.0072 (11)
C3	0.0619 (18)	0.0362 (14)	0.0423 (15)	−0.0117 (13)	−0.0133 (13)	−0.0077 (12)
C4	0.067 (2)	0.0455 (16)	0.0519 (17)	−0.0291 (15)	−0.0173 (15)	0.0016 (13)
C5	0.0536 (17)	0.0495 (16)	0.0469 (16)	−0.0240 (14)	−0.0058 (13)	−0.0009 (13)
C6	0.0489 (15)	0.0346 (13)	0.0355 (13)	−0.0114 (12)	−0.0070 (11)	−0.0001 (11)
C7	0.0473 (15)	0.0405 (14)	0.0372 (14)	−0.0113 (12)	−0.0016 (11)	−0.0053 (11)
C8	0.0508 (16)	0.0365 (14)	0.0359 (14)	−0.0124 (12)	−0.0007 (12)	−0.0059 (11)
C9	0.0490 (16)	0.0435 (15)	0.0442 (15)	−0.0146 (13)	−0.0073 (12)	−0.0045 (12)
C10	0.0531 (16)	0.0436 (15)	0.0373 (14)	−0.0198 (13)	−0.0051 (12)	−0.0047 (11)
C11	0.0524 (16)	0.0396 (14)	0.0406 (15)	−0.0113 (13)	0.0001 (12)	−0.0070 (12)
C12	0.0469 (16)	0.0486 (16)	0.0463 (16)	−0.0102 (13)	−0.0094 (13)	−0.0060 (13)
C13	0.0546 (17)	0.0455 (16)	0.0422 (15)	−0.0160 (13)	−0.0089 (13)	−0.0103 (12)

### Geometric parameters (Å, º)

**Table:**

Cl1—C10	1.733 (3)	C4—H4	0.9300
Cl2—C11	1.731 (3)	C5—C6	1.396 (4)
O1—C1	1.363 (3)	C5—H5	0.9300
O1—H1	0.8200	C6—C7	1.450 (4)
O2—C2	1.359 (3)	C7—H7	0.9300
O2—H2	0.8200	C8—C13	1.383 (4)
N1—C7	1.277 (4)	C8—C9	1.392 (4)
N1—C8	1.423 (3)	C9—C10	1.388 (4)
C1—C2	1.394 (4)	C9—H9	0.9300
C1—C6	1.401 (4)	C10—C11	1.385 (4)
C2—C3	1.375 (4)	C11—C12	1.373 (4)
C3—C4	1.390 (4)	C12—C13	1.394 (4)
C3—H3	0.9300	C12—H12	0.9300
C4—C5	1.395 (4)	C13—H13	0.9300
			
C1—O1—H1	109.5	N1—C7—C6	122.5 (3)
C2—O2—H2	109.5	N1—C7—H7	118.7
C7—N1—C8	119.6 (2)	C6—C7—H7	118.7
O1—C1—C2	117.8 (2)	C13—C8—C9	119.9 (2)
O1—C1—C6	122.1 (2)	C13—C8—N1	122.1 (3)
C2—C1—C6	120.1 (2)	C9—C8—N1	117.9 (3)
O2—C2—C3	119.1 (2)	C10—C9—C8	119.3 (3)
O2—C2—C1	120.9 (2)	C10—C9—H9	120.3
C3—C2—C1	120.0 (3)	C8—C9—H9	120.3
C2—C3—C4	120.9 (3)	C11—C10—C9	120.3 (3)
C2—C3—H3	119.6	C11—C10—Cl1	120.7 (2)
C4—C3—H3	119.6	C9—C10—Cl1	118.9 (2)
C3—C4—C5	119.5 (3)	C12—C11—C10	120.5 (3)
C3—C4—H4	120.3	C12—C11—Cl2	118.9 (2)
C5—C4—H4	120.3	C10—C11—Cl2	120.6 (2)
C4—C5—C6	120.3 (3)	C11—C12—C13	119.5 (3)
C4—C5—H5	119.8	C11—C12—H12	120.2
C6—C5—H5	119.8	C13—C12—H12	120.2
C5—C6—C1	119.3 (2)	C8—C13—C12	120.4 (3)
C5—C6—C7	119.8 (3)	C8—C13—H13	119.8
C1—C6—C7	120.9 (2)	C12—C13—H13	119.8
			
O1—C1—C2—O2	1.1 (4)	C1—C6—C7—N1	2.5 (4)
C6—C1—C2—O2	−178.3 (3)	C7—N1—C8—C13	40.5 (4)
O1—C1—C2—C3	−179.2 (3)	C7—N1—C8—C9	−143.4 (3)
C6—C1—C2—C3	1.4 (4)	C13—C8—C9—C10	−1.9 (4)
O2—C2—C3—C4	179.0 (3)	N1—C8—C9—C10	−178.1 (2)
C1—C2—C3—C4	−0.7 (4)	C8—C9—C10—C11	1.0 (4)
C2—C3—C4—C5	−0.6 (4)	C8—C9—C10—Cl1	179.0 (2)
C3—C4—C5—C6	1.3 (5)	C9—C10—C11—C12	0.5 (4)
C4—C5—C6—C1	−0.7 (4)	Cl1—C10—C11—C12	−177.5 (2)
C4—C5—C6—C7	−177.7 (3)	C9—C10—C11—Cl2	178.5 (2)
O1—C1—C6—C5	179.9 (3)	Cl1—C10—C11—Cl2	0.5 (4)
C2—C1—C6—C5	−0.7 (4)	C10—C11—C12—C13	−1.1 (4)
O1—C1—C6—C7	−3.1 (4)	Cl2—C11—C12—C13	−179.1 (2)
C2—C1—C6—C7	176.3 (3)	C9—C8—C13—C12	1.3 (4)
C8—N1—C7—C6	−172.6 (2)	N1—C8—C13—C12	177.3 (3)
C5—C6—C7—N1	179.5 (3)	C11—C12—C13—C8	0.2 (4)

### Hydrogen-bond geometry (Å, º)

Cg1 is the centroid of the benzene ring (C1–C6).

**Table:**

*D*—H···*A*	*D*—H	H···*A*	*D*···*A*	*D*—H···*A*
O1—H1···N1	0.82	1.89	2.608 (3)	146
O2—H2···O1	0.82	2.28	2.729 (3)	115
O2—H2···O1^i^	0.82	2.20	2.846 (3)	136
C12—H12···*Cg*1^ii^	0.93	2.83	3.538 (4)	134

Symmetry codes: (i) −*x*−1, −*y*+2, −*z*+1; (ii) *x*+1, *y*−1, *z*.
