# Supplementary figures and images for: Crystal structure of 3-{(E)-[(3,4-di­chloro­phen­yl)imino]­meth­yl}benzene-1,2-diol
Source: Acta Crystallogr E Crystallogr Commun. 2015 Jan 28;71(Pt 2):o137–8. doi: 10.1107/S2056989015001401 (PMC4384559; doi:10.1107/S2056989015001401)

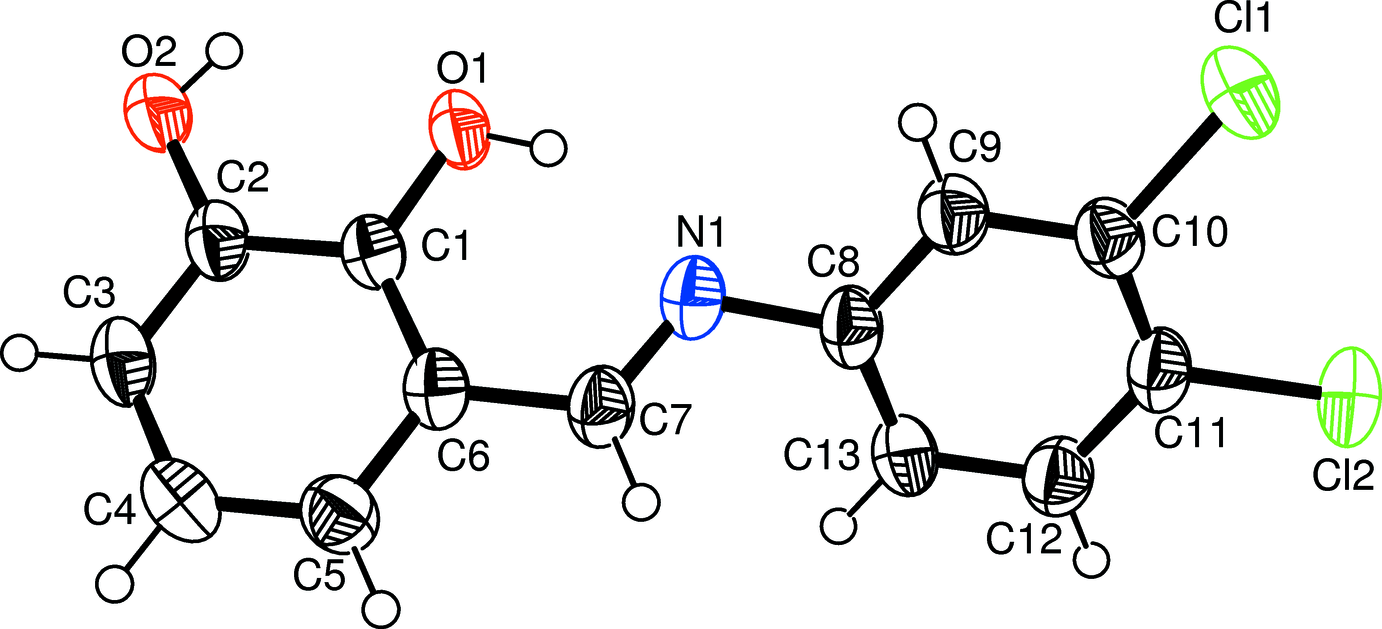

Supplement: Supplementary file 4 [file e-71-0o137-fig1.tif]

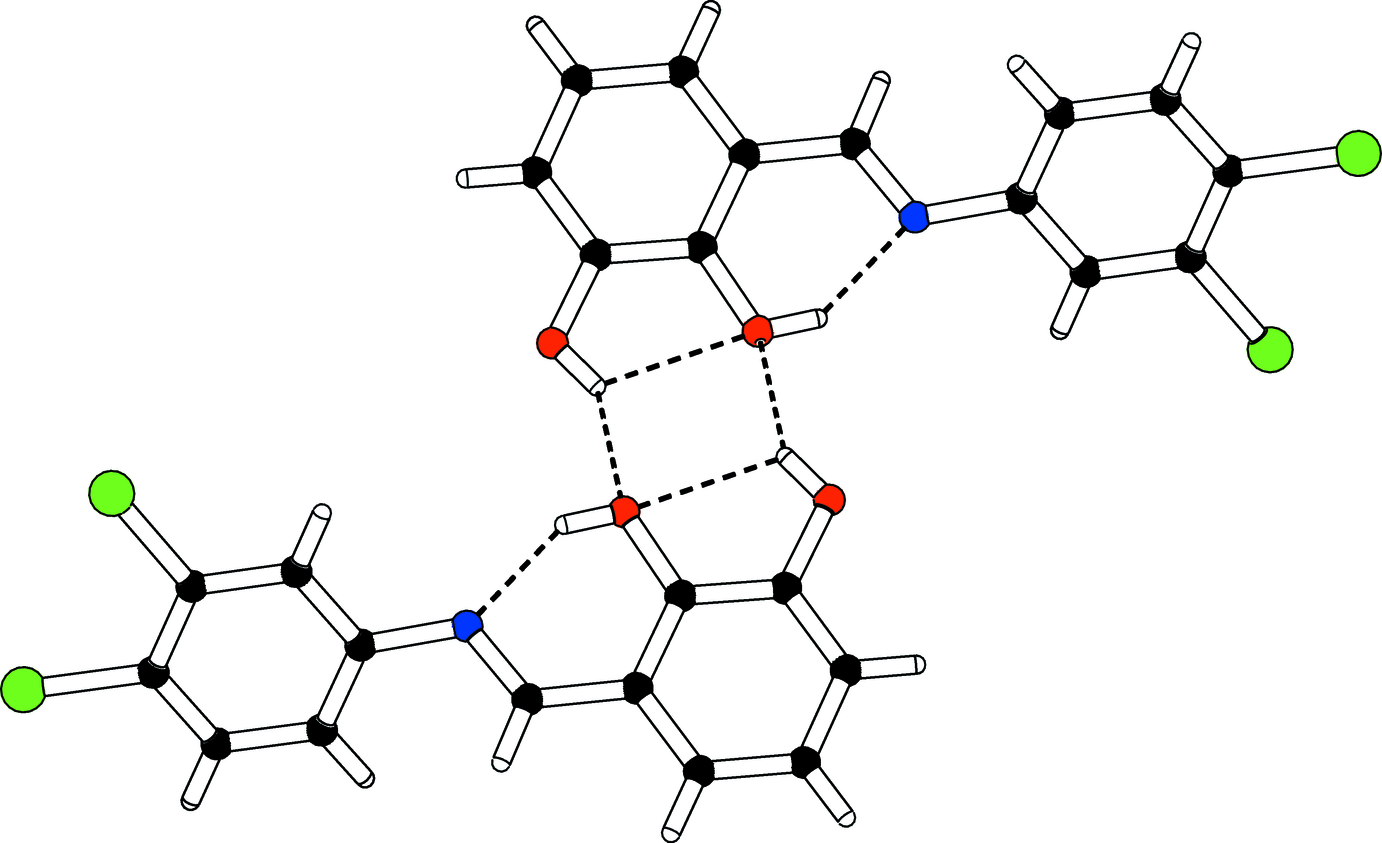

Supplement: Supplementary file 5 [file e-71-0o137-fig2.tif]
